# Dynamicity, emerging patterns, and spatiotemporal trends of scientific production on the use of activated carbon in oral health: a scientometric study

**DOI:** 10.1186/s12903-023-03375-3

**Published:** 2023-09-16

**Authors:** Frank Mayta-Tovalino, Fran Espinoza-Carhuancho, Daniel Alvitez-Temoche, Cesar Mauricio-Vilchez, Arnaldo Munive-Degregori, John Barja-Ore, Josmel Pacheco-Mendoza

**Affiliations:** 1 Vicerrectorado de Investigación, Universidad San Ignacio de Loyola, Lima, Peru; 2 Grupo de Bibliometría, Evaluación de evidencia y Revisiones Sistemáticas (BEERS), Human Medicine Career, Universidad Cientifica del Sur, Lima, Peru; 3 Academic Department, Faculty of Dentistry, Universidad Nacional Federico Villarreal, Lima, Peru; 4 Academic Department, Universidad Nacional Mayor de San Marcos, Lima, Peru; 5 Direction of Research, Universidad Privada del Norte, Lima, Peru; 6 Dirección General de Investigación, Desarrollo e Innovación, Universidad Científica del Sur, Lima, Peru

**Keywords:** Activated carbon, Bibliometrix, Oral health, Scientometric study, Web of Science

## Abstract

**Background:**

The use of activated carbon (AC) in oral hygiene products has gained significant interest; however, its potential benefits for oral health remain uncertain. This study aimed to conduct a scientometric analysis to examine the dynamicity, emerging patterns, and trends over time in scientific production concerning the use of AC in oral health.

**Methods:**

The Web of Science database was searched for articles published between 2005 and 2022. Various bibliometric indicators, including the H-index, annual growth, Lotka’s law, Bradford’s law, and Sankey diagram, were used for data analysis. Overlay maps, timezone visualization, and three field plots were used to evaluate visualization patterns, time–temporal relationships, and trends. Information retrieval process was performed on March 11, 2023.

**Results:**

The analysis revealed that only six studies constituted the top references with the highest number of citations in recent years, with Brooks’ 2017 study demonstrating the most significant increase in citation. The dual-map overlay demonstrated a close citation relationship between cluster 4 (Molecular Biology Immunology) and the areas of Environmental, Toxicology, and Nutrition. The visualization graph of publication patterns indicated the journals that accumulated the highest number of citations during the study period.

**Conclusion:**

This scientometric study provides valuable insights into the use of AC in oral health and its impact on the field of dentistry. It determines the most productive journals, authors, and countries with the greatest influence. AC effectively removes pollutants and is gaining interest for use in dental effluent treatment. Thus, it may be a viable option for professionals.

## Background

Activated carbon (AC)-containing oral hygiene products used to remove dental stains and lighten teeth are becoming increasingly popular in various international markets [1]. The efficacy of whitening toothpastes containing AC is controversial, as some in vitro studies found that continuous use of such toothpastes is effective compared with the use of conventional toothpastes [2]. However, other studies found no improvement in tooth color with the use of powdered AC [3, 4] or toothpastes containing AC [5–7].

To date, the impact of AC-containing products on the enamel surface is uncertain, as in vitro studies present conflicting findings. Some studies have reported an increase in enamel surface roughness with the use of AC [3, 5, 7], whereas others have reported no significant changes [4–6]. In the field of endodontics, silver-impregnated AC has shown promise in removing volatile compounds and by-products from chemomechanically prepared infected root canals in certain in vitro studies [7]. However, further research is warranted to validate these observations. AC-containing mouthwashes have gained popularity for their potential antimicrobial, anti-halitosis, clarifying, remineralizing, anticaries, and periodontal disease control properties. Nevertheless, the efficacy of these actions lacks sufficient evidence [8].

Overall, there is a paucity of evidence of the efficacy of various types of AC-containing products. This should be taken into account by clinicians when recommending such products to patients, particularly considering studies that indicate the potential undesired effects of these products, such as the ability of carbon to absorb fluoride and reduce its efficacy [9]. Furthermore, it is noteworthy that many dental professionals, particularly in developing countries, are unfamiliar with dental products containing AC, including their properties, uses, and indications. This highlights the need for further information and education on these products to promote their widespread use [10].

Due to the scarcity of robust scientific evidence, there is still a lack of studies to be able to meta-analyze them. This study aimed to conduct a scientometric analysis to evaluate the dynamicity, emerging patterns, and spatiotemporal trends of scientific production on the use of AC in oral health.

## Methods

### Study design

A descriptive and observational scientometric study was conducted using the Web of Science database (Core Collections) to analyze published research related to the topic. The search strategy was formulated using MESH (Medical Subject Headings) and Emtree terms (Embase). The search covered the period of 2005–2023.

### Search strategy

All published articles on the subject were included, without any restriction by publication date or language. The downloaded metadata [*n* = 35] were saved in plain text format and csv [comma separated value] format. The study was conducted on March 11, 2023, involving a search for information in Web of Science, a comprehensive reference source that covers a wide range of specialized publications in the field of health. Web of Science was used as it is one of the most prestigious multidisciplinary databases, which guarantees the quality of its content. It also allows an advanced analysis of scientometric metrics and an advanced search of information.

To conduct the research, the MESH thesaurus was used, and a search strategy was defined using the logical operators “AND” and “OR.” The key aspects of the selected search strategy are described in detail in the following sections: TS = [“Activated charcoal” OR “Activated carbon” OR “Activated carbon filter” OR “Activated carbon powder” OR “Activated carbon pellets” OR “Activated carbon granules” OR “Steam activated carbon” OR “Acid-washed activated carbon” OR “Base-washed activated carbon” OR “Coconut shell activated carbon” OR “Wood-based activated carbon” OR “Carbon adsorbent” OR “acta-char” OR “activated coal” OR “active carbon” OR “active charcoal” OR “active coal” OR “black coal” OR “brominated activated carbon” OR “carbolen” OR “carbon active” OR “charcocaps” OR “super char”] AND TS = [“Toothpaste” OR “Dental cream” OR “Tooth gel” OR “Oral gel” OR “Teeth cleaning paste” OR “Dentifrice” OR “crest mint” OR “crest regular” OR “dental powder” OR “dentifrice*” OR “fresh breath” OR “orabase” OR “plus white” OR “sensodyne” OR “thermodent” OR “tooth paste*” OR “toothpaste*” OR “ultrabright” OR “Walgreen” OR “worthmore” OR “Mouth Rinse*” OR “Rinse Mouth” OR “Mouth Bath” OR “Bath Mouth” OR “Mouth Wash*” OR “Wash Mouth” OR “Colgate Plax Overnight” OR “mouthrinse*” OR “mouthwash*”].

### Bibliometric indicators

In this study, various production, collaboration, and impact indicators were used, including the H-index, annual growth, Lotka’s law, Bradford’s law, and Sankey diagram. Thematic maps were used to visualize author and country relationships as well as collaborations. In addition, analytical graphs such as timezone visualization, citation burst, and overlay maps were used to examine dynamics and spatiotemporal patterns.

### Data analysis

After downloading the data, they were processed in the Mendeley software to check for duplicates. Subsequently, the analyzed metadata, including author name, citations received, journal, and country, were exported to CiteSpace 6.2 R2 [2003–2023 Chaomei Chen] and Bibliometrix 3.0 and Bibliometrix 3.0. Finally, they were exported to CiteSpace 6.2 R2 [2003–2023 Chaomei Chen] and Bibliometrix 3.0 R version 4.2.3 [2023-03-15 ucrt].

## Results

During the period of 2001–2023, a total of 35 manuscripts were identified in the study. The annual growth rate was calculated as 7.59%, with the average document age being 3.3 years. Each document received an average of 6,229 citations, and a total of 1,122 references were cited. The research involved 170 authors, and there were no single-authored papers. On average, there were 5.09 co-authors per paper, and 17.14% of these collaborations were international (Table 1).

**Table 1:** Scholarly Output

Description	Results
Timespan	2001–2023
Sources [Journals, Books, etc]	27
Documents	35
Annual Growth Rate %	7.59
Document Average Age	3.3
Average citations per doc	6.229
References	1122
**Document contents**	
Keywords Plus [ID]	110
Author’s Keywords [DE]	121
**Authors**	
Authors	170
Authors of single-authored docs	0
**Authors collaboration**	
Single-authored docs	0
Co-Authors per Doc	5.09
International co-authorships %	17.14
**Document types**	
Article	28
Article; early access	2
Review	5

**Fig. 1:**
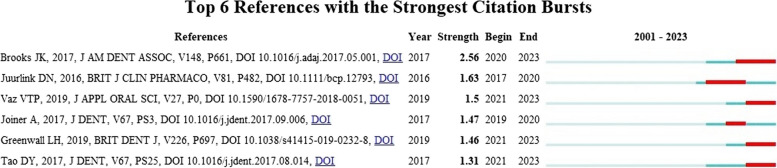
Top references with the strongest citation bursts

Among the identified studies, six stood out as the top references with the strongest citation bursts in recent years. The study by Brooks in 2017 had the highest burst, with a strength of 2.56 during the period of 2022–2023. It was followed by Juurlink’s study in 2016, with a burst of 1.63, and then Tao’s study in 2017, with a burst of 1.31 (Fig. 1).

**Fig. 2:**
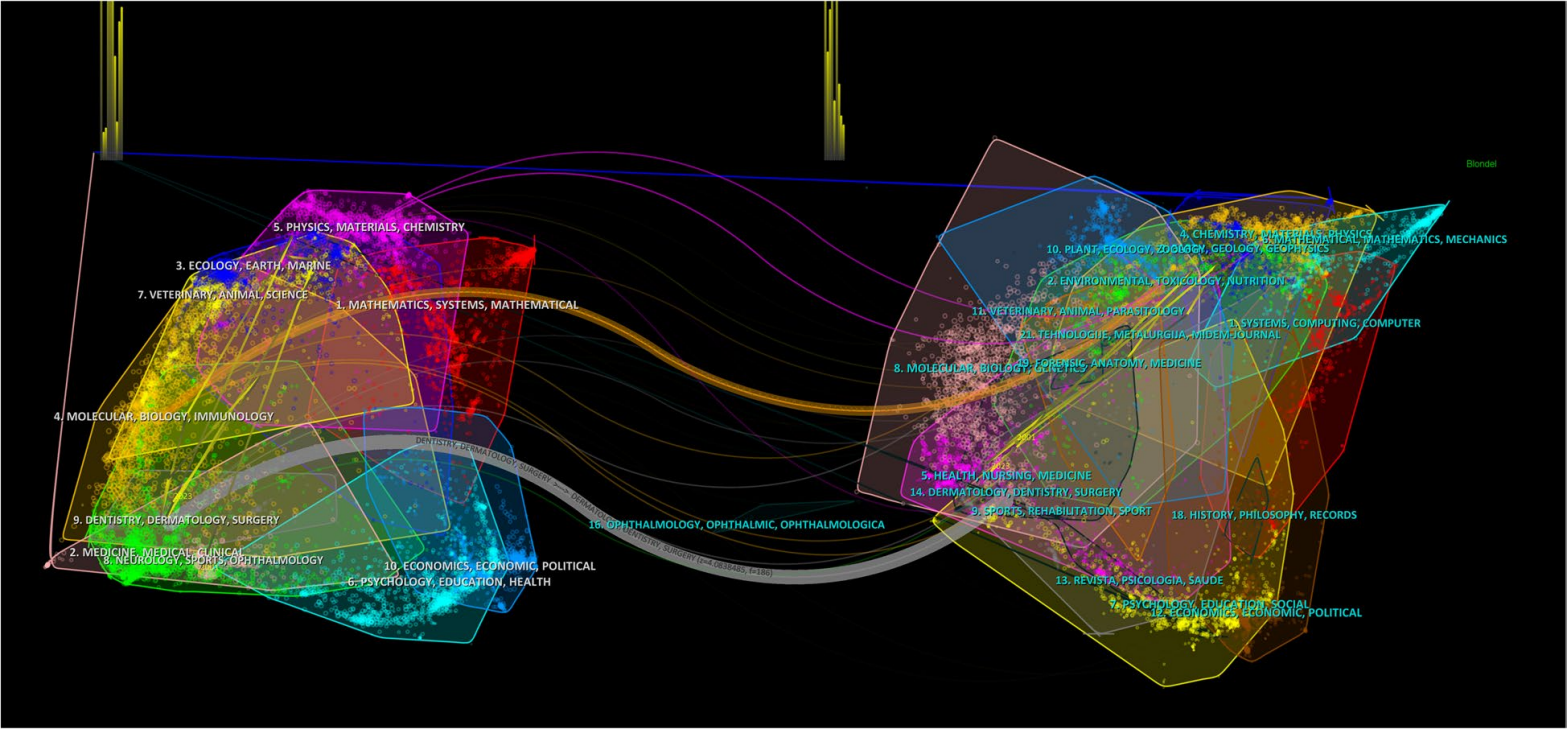
Overlay maps

The dual-map overlay provides an overview of the disciplines involved, extracted from the analyzed journals, with citation links depicted from left to right, showing the citation patterns and their impact on the respective areas. The prominent orange line indicates a significant citation relationship between cluster 4 (Molecular Biology Immunology) and the areas of Environmental, Toxicology, and Nutrition. In addition, the thicker lead line indicates a stronger relationship between cluster 9 (Dentistry, Dermatology, Surgery), which received more citations in research from the thematic fields of Health, Nursing, and Medicine. These lines show the relevance, flow, and connection between these knowledge domains (Fig. 2).

**Fig. 3:**
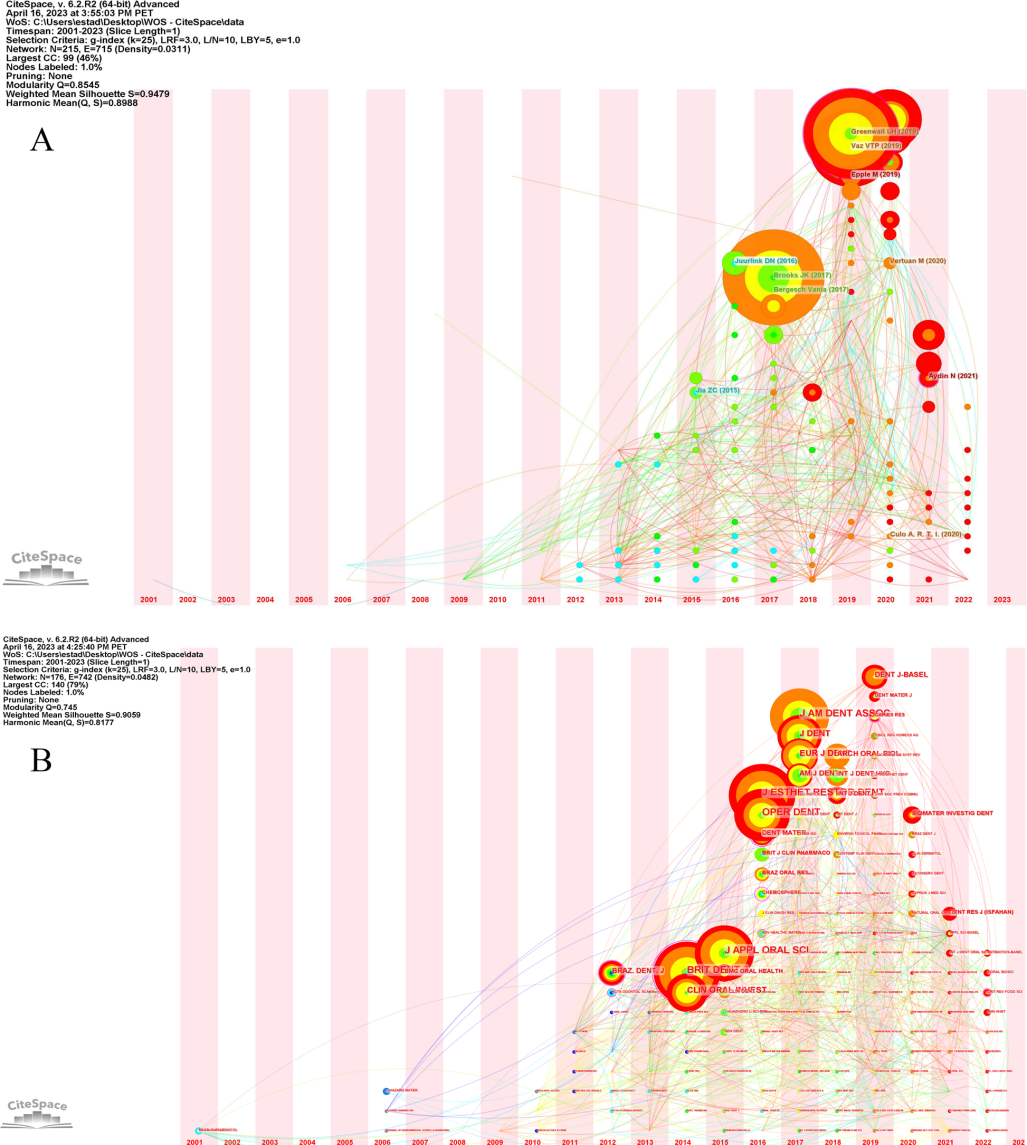
Timezone visualization

Figure 3 A, showing the timezone visualization, demonstrates that the largest nodes appeared in 2017 and 2019, represented by authors such as Brooks (2017), Juurlink (2016), and Greenwall and Vaz (2019). These nodes indicate multiple authors distributed over time, signifying continuity in the citation of the evaluated authors.

In Fig. 3B, the timezone visualization graph provides insights into the publication patterns of journals that accumulated the most citations in recent years. Clin Oral Inv, Brit Dent, and J Appl Oral Sci had the largest nodes, corresponding to 2014 and 2015. In addition, a significant citation activity was observed between 2016 and 2019. This analysis revealed the citation patterns and trends in the international collaboration and spatial distribution of scientific production on AC in dentistry.

**Fig. 4:**
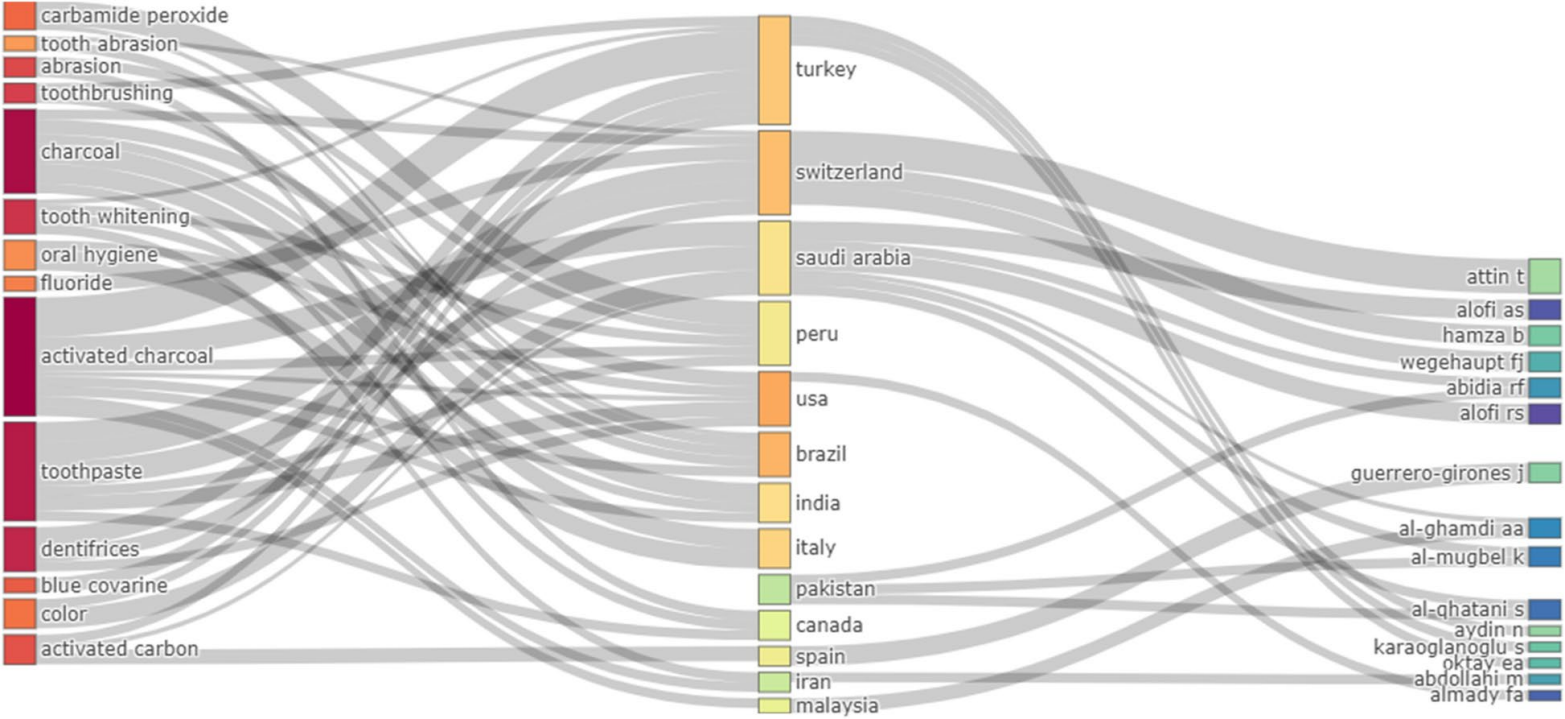
Three-field plot

The Sankey diagram (three-field plot) illustrates the flow of relationships normalized by keyword–country–author. The larger rectangular nodes in each category allow for visual evaluation of the relationships among the evaluated elements. The color intensity and size of the rectangle indicate strong relationships between keywords, such as “charcoal,” “activated charcoal,” “toothpaste,” and countries like “Turkey,” “Switzerland,” and “Saudi Arabia,” as well as authors like “Atiin t” and “Wegehaupt fj” (Fig. 4).

**Fig. 5:**
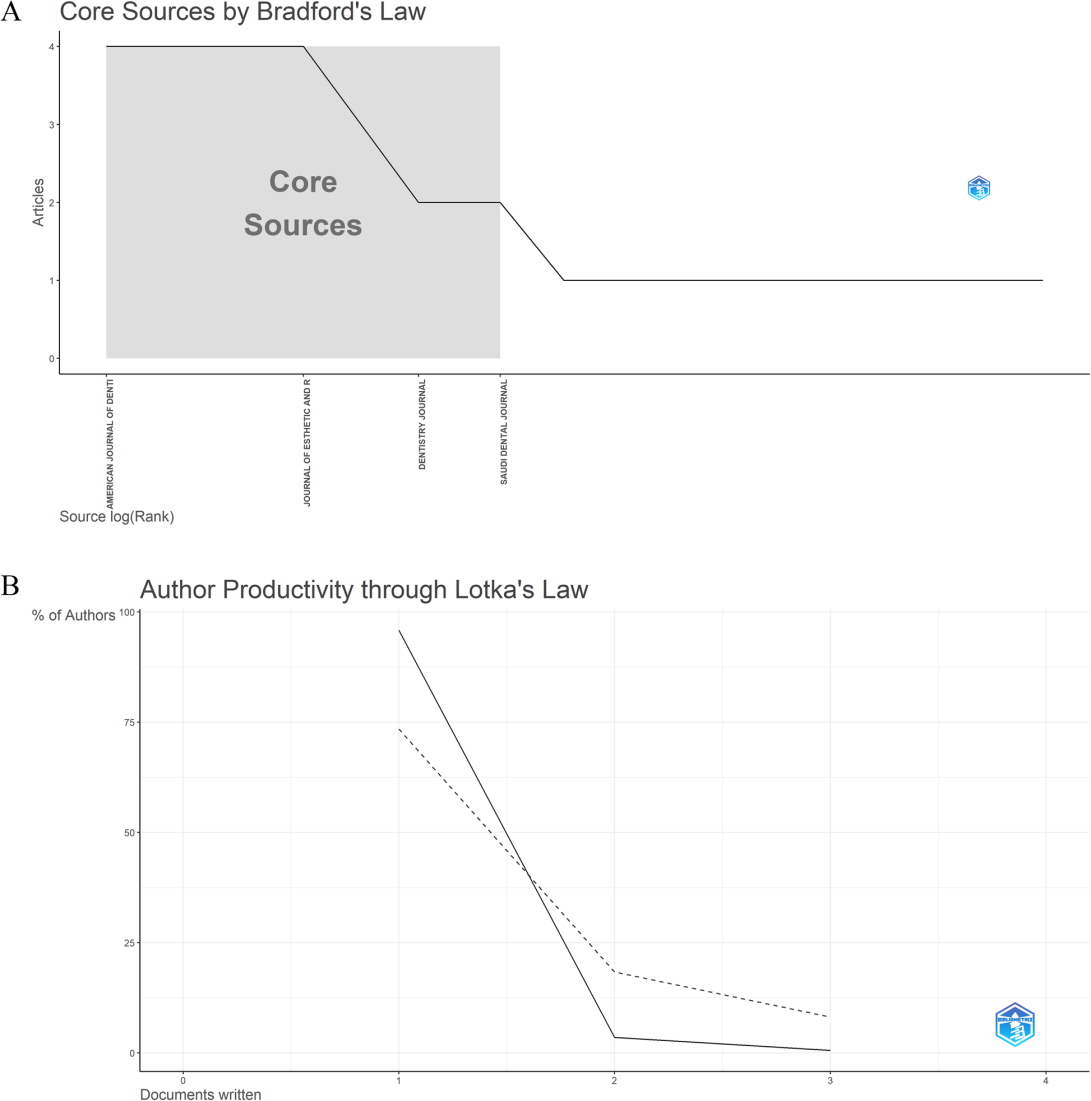
Bradford’s law and Lotka’s law

Analysis of core sources using Bradford’s law revealed the most relevant journals in the distribution of scientific publications. As can be seen from Fig. 5A, most of the publications were concentrated on the first zone, with four main journals (Am J Dent, J Esthet Restor Dent, Dent J, and Saudi Dent J) being particularly important. This indicates that these four journals play a crucial role in the dissemination of research on the topic, although other relevant sources exist.

The application of Lotka’s law to author productivity revealed that around 95% of the manuscripts were written by authors with only one published document, whereas approximately 3% were written by authors with two published documents. This analysis revealed that most authors in this field are not highly productive, with a small group being responsible for a significant portion of the publications (Fig. 5B).

**Fig. 6:**
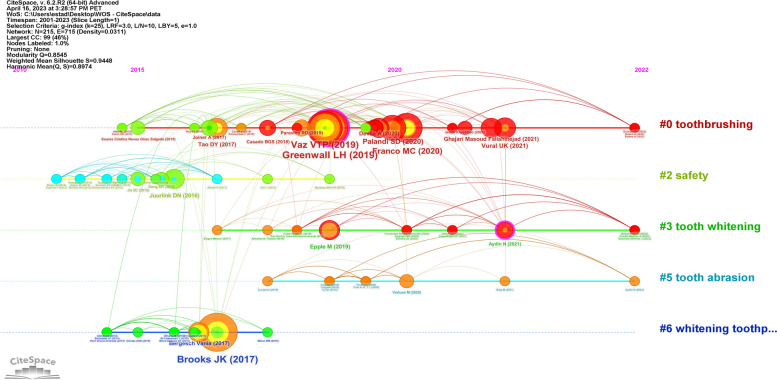
Timeline view clusters

The timeline view cluster graph identified six major lines corresponding to the most important clusters, including “toothbrushing,” “safety,” “tooth whitening,” “tooth abrasion,” and “whitening toothpaste.” The larger nodes indicate the most cited references in each timeline and corresponding years. Active co-citation is indicated by the strong interrelation between links and different nodes. Notably, Greenwall (2019) and Vaz VTP (2019) received high citations in the cluster of “toothbrushing,” whereas Brook (2017) led the research on “whitening toothpaste” since 2015. This demonstrates the temporal evolution of trends and collaboration patterns in scientific publication citations (Fig. 6).

**Fig. 7:**
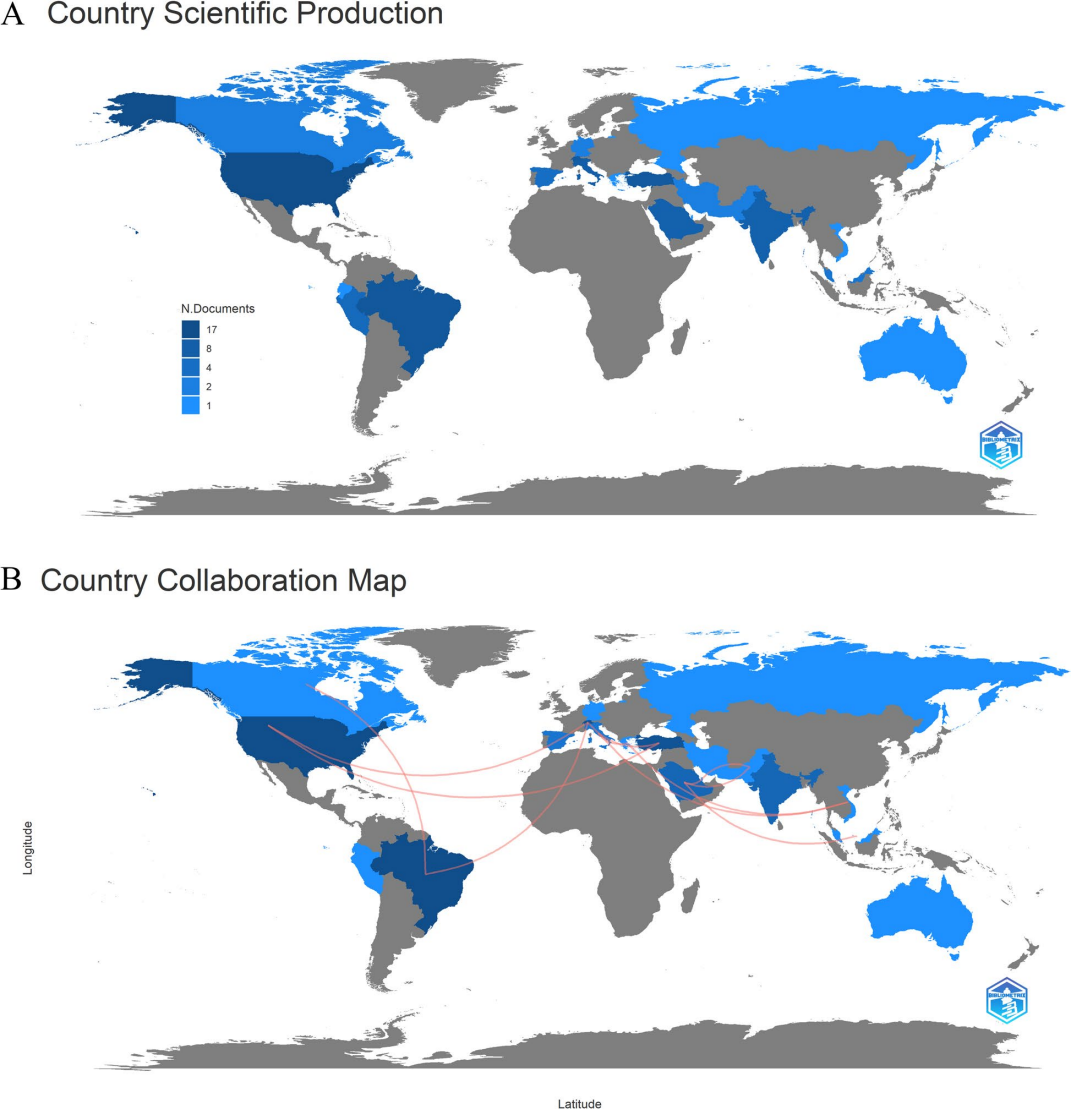
Country scientific and collaboration map

Regarding the bibliometric analysis of scientific production by country, the USA had the highest frequency of publications, which was 17, followed by Brazil and Switzerland, with 11 and 10 publications, respectively. Other countries such as India and Saudi Arabia had notable presence with seven publications each, whereas Peru, Spain, and Malaysia had five, four, and three, respectively (Fig. 7A). The world collaboration map highlighted several instances of international collaboration, including Brazil’s collaborations with Canada and Switzerland, Greece’s collaboration with Vietnam, and Italy’s collaborations with Greece and Vietnam. Notably, there was a collaboration between Pakistan and Qatar, as well as Saudi Arabia’s collaborations with Malaysia, Pakistan, and Qatar. It is noteworthy that while collaboration is valuable, it should not be the sole indicator for quantifying collaborative networks (Fig. 7B).

## Discussion

Due to the increased visibility of AC-containing products in the commercial market, it is necessary for oral healthcare professionals to provide guidance on their usage. However, it is important to note that there is currently insufficient scientific evidence on the safety and efficacy of these products, both in cosmetic and therapeutic applications [8].

This study highlights the prominence of the research areas of Health, Nursing, and Medicine in the production of research on AC use. This trend may be attributed to the growing popularity and acceptance of natural products for oral healthcare, as reported by Mathias-Santamaria et al. [9]. Six studies were identified as key references, exhibiting a significant increase in citations in recent years. For instance, Brooks’ study in 2017 had the highest increase [strength 2.56], followed by Juurlink’s study in 2016 [strength 1.63] and then Tao’s study in 2017 [strength 1.31].

Among the influential authors in this research area, Vaz stood out as a significant contributor. One of his studies showed the effective whitening effect of AC, although other technologies, such as hydrogen peroxide and abrasive microspheres, have been found to be more effective [2]. Conversely, Franco et al. [10] concluded that powdered AC does not exert a whitening effect.

The analyzed publications were published in high-impact scientific journals, thus ensuring greater visibility and reach within the scientific and academic community interested in this field. This should promote evidence-based improvements in professional practice. However, it is noteworthy that in certain contexts, dental professionals have limited knowledge and awareness of the impact of AC-containing products [11].

Country-based analysis revealed that Brazil significantly contributes to the scientific production on AC in oral health, actively collaborating with other countries such as Canada and Switzerland. Similarly, da Silva Santos et al. [12] identified Brazil as the leading Latin American country in this domain.

Therefore, it is important to recognize that biochar represents a multidisciplinary research opportunity with diverse applications [13, 14]. Collaboration with research teams from different institutions and countries is necessary to enhance and expand scientific evidence, leading to positive impacts on professional practice and people’s health.

Analysis of research lines revealed that the primary areas of focus were “tooth brushing,” “safety,” “tooth whitening,” “tooth abrasion,” and “whitening toothpaste” [3, 5, 15]. Given this, dental professionals are recommended to collaborate with other experts to bridge the gap in scientific evidence through studies that enhance people’s quality of life. Bibliometrics, as a broad field of study applied to various scientific disciplines, offers different and innovative metric indicators for evaluating the impact of citations on authors’ H-index, which should be considered for a comprehensive analysis [16, 17]. Based on the available evidence, it can be mentioned that AC has been used in various dental treatments owing to its diverse properties in toxin and enamel stain elimination. However, it is noteworthy that robust clinical evidence specifying its efficacy is still lacking. Therefore, it is recommended to continue conducting scientific research to further explore its efficacy [7–13, 18].

In summary, these findings should be interpreted with caution as the use of AC in oral health is still an evolving area. Finally, among the identified studies, there is evidence suggesting a potential positive effect of AC on oral health.

## Conclusions

Within the limitations of this exploratory scientometric study, six studies were identified that stood out for their high number of citations and strength in recent years. Brooks (2017) and Juurlink (2016) were the authors with the highest impact. The study also identified the disciplines most closely associated with the citation patterns, demonstrating a strong correlation between the fields of health, nutrition, molecular biology, and immunology. Furthermore, it was evidenced that 2017 and 2019 were the years with the highest number of nodes and authors connected in the citation network. Evidence shows that AC is potentially effective in removing a wide variety of pollutants. There is also a growing interest in its use in effluent dental treatment. Therefore, it could be a viable option to suggest to professionals.

## Data Availability

All the data presented in this manuscript are available in the Scopus database using the search query in the methodology section. In addition, the data used during the current study are available from the corresponding authors can be requested from the corresponding author.

## Abbreviation

AC: Activated carbon
